# β-alanine supplementation improves YoYo intermittent recovery test performance

**DOI:** 10.1186/1550-2783-9-39

**Published:** 2012-08-28

**Authors:** Bryan Saunders, Caroline Sunderland, Roger C Harris, Craig Sale

**Affiliations:** 1 Sport, Health and Performance Enhancement (SHAPE) Research Group, School of Science and Technology, Nottingham Trent University, Clifton Lane, Nottingham, NG11 8NS, UK; 2 Junipa Ltd, Newmarket, Suffolk, UK

**Keywords:** β-alanine, Carnosine, YoYo Intermittent recovery test, Repeated sprint activity, Team sport specific exercise capacity

## Abstract

**Background:**

β-alanine supplementation has been shown to improve high-intensity exercise performance and capacity. However, the effects on intermittent exercise are less clear, with no effect shown on repeated sprint activity. The aim of this study was to investigate the effects of β-alanine supplementation on YoYo Intermittent Recovery Test Level 2 (YoYo IR2) performance.

**Methods:**

Seventeen amateur footballers were allocated to either a placebo (PLA; N = 8) or β-alanine (BA; N = 9) supplementation group, and performed the YoYo IR2 on two separate occasions, pre and post 12 weeks of supplementation during a competitive season. Specifically, players were supplemented from early to mid-season (PLA: N = 5; BA: N = 6) or mid- to the end of the season (PLA: N = 3; BA: N = 3). Data were analysed using a two factor ANOVA with Tukey post-hoc analyses.

**Results:**

Pre supplementation scores were 1185 ± 216 and 1093 ± 148 m for PLA and BA, with no differences between groups (P = 0.41). YoYo performance was significantly improved for BA (+34.3%, P ≤ 0.001) but not PLA (−7.3%, P = 0.24) following supplementation. 2 of 8 (Early – Mid: 2 of 5; Mid – End: 0 of 3) players improved their YoYo scores in PLA (Range: -37.5 to + 14.7%) and 8 of 9 (Early – Mid: 6 of 6; Mid – End: 2 of 3) improved for BA (Range: +0.0 to +72.7%).

**Conclusions:**

12 weeks of β-alanine supplementation improved YoYo IR2 performance, likely due to an increased muscle buffering capacity resulting in an attenuation of the reduction in intracellular pH during high-intensity intermittent exercise.

## Introduction

Carnosine (β-alanyl-L-histidine) is a naturally occurring dipeptide found in high concentrations in skeletal muscle [1] and due to its pKa (6.83), it is a suitable buffer over the exercise intramuscular pH transit-range [2]. β-alanine supplementation has been shown to be effective in increasing muscle carnosine levels [1], thereby increasing muscle buffering capacity, with the potential to improve exercise performance and capacity that is limited by the accumulation of hydrogen ions (H^+^) [3,4]. Recent research has focussed on repeated sprint ability, a key component of team sport performance [5], due to its association with H^+^ buffering capacity in both professional and amateur footballers [6]. Despite this, research has shown no effect of β-alanine supplementation on repeated sprint performance alone [7,8], or repeated sprints performed during simulated games play [9]. However, these protocols measure high-intensity exercise performance of less than 60 s in duration and, in a meta-analysis of the literature, Hobson et al. [10] showed that β-alanine was most effective in improving exercise capacity during exercise lasting in excess of 60 s. Therefore, β-alanine supplementation may be more effective in increasing sport specific high-intensity intermittent exercise capacity.

The YoYo Intermittent Recovery Tests (Level 1 [YoYo IR1] and 2 [YoYo IR2]) were designed [11] to evaluate the ability of an individual to repeatedly perform and recover from intense exercise, and are applicable to team sports players due to the specificity of the exercise undertaken [12]. These tests have been shown to be sensitive to training adaptations [13,14], seasonal variation [13] and differences in playing position and playing standard [13,15]. Furthermore, YoYo Intermittent Recovery Test performance is closely related to football match performance, since YoYo IR1 outcomes are correlated with high intensity running and total distance covered during a football match for top class referees [16] and footballers [13]. The highest distance covered in a 5 min period during a game has also been associated with YoYo IR2 performance [12]. These findings suggest that the YoYo IR Tests are appropriate models for examining the effects of interventions designed to manipulate changes in individual performance during team sport exercise.

Football is a sport that requires players to perform substantial high-intensity running with a large contribution from both aerobic and anaerobic energy pathways. The YoYo IR2 best evaluates an individual’s capacity to perform repeated high-intensity exercise while simultaneously stimulating both aerobic and anaerobic energy systems [13]. At volitional exhaustion, muscle lactate and glycogen utilisation are higher, and muscle pH is lower, following the YoYo IR2 compared to the YoYo IR1 test [12], suggesting a larger activation of the anaerobic energy system towards the end of the YoYo IR2. Interestingly, muscle pH was significantly decreased (and muscle lactate increased) at exhaustion compared with at 85% exhaustion time, while muscle phosphorylcreatine and glycogen were not [13]. This indicates that decreased muscle pH may be a significant contributing factor to fatigue during the YoYo IR2, suggesting that the YoYo IR2 is a suitable model to investigate the effect of increased muscle buffering capacity on team sport specific fitness.

No study has examined the effects of supplementation on team sport specific exercise capacity. Therefore, the aim of this investigation was to examine the effect of β-alanine supplementation on YoYo IR2 performance in well-trained amateur footballers throughout a competitive season. We hypothesised that β-alanine would significantly improve the distance covered during the test due to an increase in intracellular pH buffering as the result of muscle carnosine elevation.

## Methods

### Subjects

Seventeen amateur male footballers (age 22 ± 4 y, height 1.83 ± 0.06 m, mass 76.9 ± 6.6 kg) from the same club competing in the lower divisions of the English football pyramid volunteered for the study and were randomly allocated to either a placebo (PLA) or β-alanine (BA) group. All players were members of the same team and were engaged in an identical team sport specific training regime over the season. Health screening, using questionnaires, was repeated prior to each laboratory visit to ensure the health status of the subjects had not changed. Subjects had not taken any supplement in the 3 months prior to the study and had not taken β-alanine for at least 6 months. None of the subjects were vegetarian and, therefore, would have encountered small amounts of β-alanine in their diet from the hydrolysis of carnosine and its methyl derivatives in meat. The study was approved by the institution’s Ethical Advisory Committee.

### Study design

All subjects had performed the YoYo IR2 on a minimum of two previous occasions, and were aware of the requirements of the protocol. Subjects were required to perform the YoYo IR2 on two separate occasions, separated by 12 weeks of supplementation. Subjects maintained a food diary in the 24 h period before the first main trial, and this was subsequently used to replicate the diet prior to the second main trial.

Subjects were supplemented with either 3.2 g·day^-1^ of β-alanine (CarnoSyn^TM^, NAI, USA) or placebo (maltodextrin, NAI, USA), provided in the form of 800 mg sustained-release tablets, over a 12 week period. Players were supplemented from early to mid-season (PLA: N = 5; BA: N = 6) or mid- to the end of the season (PLA: N = 3; BA: N = 3). There were no differences in YoYo IR2 performance prior to supplementation between players starting early season and mid-season for either group (PLA: P = 0.38, 1128 ± 241 and 1280 ± 160 m; BA: P = 0.48, 1120 ± 143 and 1040 ± 174 m). The dosing regimen consisted of one 800 mg β-alanine or placebo tablet ingested four times per day at 3 – 4 h intervals. Compliance with the supplementation regimen was monitored using supplementation logs, with a high degree of compliance being reported in both groups (Placebo: 89%; β-alanine: 90%). There were no reports of symptoms of paraesthesia from any of the subjects in either group. All supplements were tested by HFL Sports Science prior to use to ensure no contamination with steroids or stimulants according to ISO 17025 accredited tests.

### YoYo intermittent recovery test level 2

All tests were performed indoor on an artificial running track in ambient conditions (temperature 21.0 ± 0.7°C, relative humidity 52.4 ± 0.8%). Upon arrival, subjects performed a 5 min standardised warm-up, consisting of light jogging and running, followed by 5 min of self-selected stretching. The YoYo IR2 consists of repeated 40 m (2 x 20 m) runs between markers set 20 m apart, at progressively increasing speeds dictated by an audio signal [11]. Subjects perform 10 s of active recovery between each running bout, consisting of a 10 m (2 x 5 m) walk. The test was ended if the player failed to reach the finish line within the given time frame on two consecutive occasions or if the player felt unable to continue (volitional exhaustion). The total number of levels was recorded and used to determine total distance covered (m) during the test.

### Statistical analysis

All data were analysed using Statistica 9 (Statsoft, USA) and are presented as mean ± 1SD. A two factor ANOVA (Group x Trial) was used to determine any differences in YoYo performance. Tukey tests were used for post-hoc analyses and effect sizes were calculated using Cohen’s d [17]. Statistical significance was accepted at the P ≤ 0.05 level.

## Results

**Figure 1:**
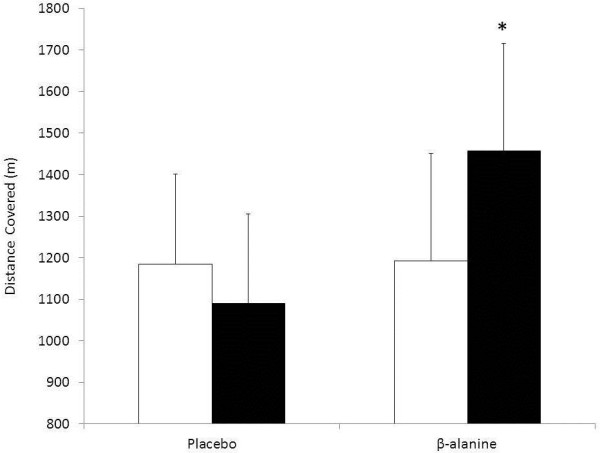
**Distance covered during the YoYo IR2 for both supplementation groups pre (white bars) and post (black bars) supplementation.** *P ≤ 0.001 from pre supplementation.

There was no significant difference in the distance covered during the YoYo IR2 (P = 0.83; PLA: 1185 ± 216 m and BA: 1093 ± 148 m, d = 0.54) between the placebo and β-alanine groups prior to supplementation. There was a significant interaction effect (Group x Trial, P ≤ 0.001), with no difference for PLA (−7.6 ± 16.2%; *post hoc* P = 0.62, d = 0.43) and a significant improvement for BA (+34.3 ± 22.5%; *post hoc* P ≤ 0.001, d = 1.83) following supplementation (Figure 1).

**Figure 2:**
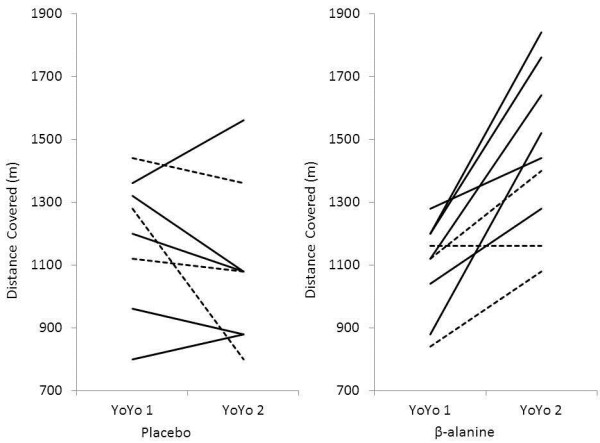
**Individual response to supplementation in the placebo and β-alanine groups pre (YoYo 1) and post (YoYo 2) supplementation.** Players supplemented from early to mid-season are indicated by a solid line and players supplemented from mid- to the end of the season are indicated by a dotted line.

Performance changes ranged from −37.5 to + 14.7% in PLA, and +0.0 to +72.7% in BA. In total, 2 of the 8 players in PLA showed an improvement in performance, with the remaining subjects having a reduction in performance from −40 to −480 m. In comparison, 8 out of 9 players showed improvement in BA (+160 to +640 m), with the remaining player unchanged (Figure 2). Subject 17 in the BA group showed an unusually high increase in YoYo IR2 performance (+72.7%) given that the response usually shown in response to pre-season training is 42%. Due to this, we removed this subject and then reanalysed the data, which did not change any of the study outcomes (Group x Trial, P = 0.001; BA: +29.4 ± 18.4%, *post hoc* P = 0.003).

In the group of players supplemented from early to mid-season, 2 out of 5 in PLA and 6 out of 6 in BA group improved YoYo IR2 performance. Of the remaining players supplemented from mid until the end of season, no one in PLA showed an improvement while 2 out of 3 in BA improved their distance covered.

## Discussion

There was a clear effect of 12 weeks of β-alanine supplementation on the distance covered during the YoYo IR2 test. This is in contrast to previous research that has shown no effect of β-alanine on repeated sprint exercise [7-9], although these studies used exercise protocols consisting of performance tests incorporating periods of high-intensity and sprint activity of less than 60 s in duration, which are suggested to be unaffected by β-alanine supplementation [10]. The YoYo IR2 is an exercise capacity test designed to last between 5 and 15 minutes and aims to evaluate an individual’s ability to perform repeated bouts of high-intensity exercise with a large contribution from anaerobic energy sources. Furthermore, distance covered during the YoYo IR2 has been associated with high-intensity running performed during competitive games play [12,13]. Therefore, the results of the present investigation suggest that β-alanine supplementation is effective at improving team sport specific exercise capacity.

Blood measures were not taken in the current investigation, although others have reported lactate values in excess of 10 mmol·L^-1^ at exhaustion [13], which is higher than the values shown in repeated sprint activity studies that have shown a correlation to H^+^ buffering capacity (~8 mmol·L^-1^; [5,18]). Although the rate of muscle phosphorylcreatine and glycogen utilisation are high during the YoYo IR2 [13], there is no difference in muscle concentrations of these substrates between 85% and 100% of exhaustion time, indicating that depletion of these substrates is not a main contributing factor to fatigue. Interestingly, muscle pH was significantly lower at exhaustion compared with at 85% of exhaustion time, which suggests increasing muscle acidity is a limiting factor to YoYo IR2 performance. Although muscle carnosine concentrations were not directly determined in this study, Stellingwerff et al. [19] showed that as little as two weeks of β-alanine supplementation at half the dose used in the current study was sufficient to increase muscle carnosine by 11.8 ± 7.4% in the *tibialis anterior*. Therefore, it can by hypothesised that 12 weeks of β-alanine supplementation at 3.2 g·day^-1^ significantly increased muscle carnosine concentrations in the current population. As such, since one of the undisputed roles of muscle carnosine is in muscle buffering, the most likely explanation for the improvement in YoYo IR2 performance is due to an increase in intracellular buffering capacity, resulting in an attenuation of the reduction in intracellular pH during high-intensity exercise.

The YoYo IR2 has been shown to be a highly reproducible capacity test, with a CV of ~10% for two tests performed within a one week period [13]. In addition, the test is sensitive to detect training adaptions, with performance improvements of approximately 42% shown following pre-season training. In the present investigation, players in the placebo group showed a ~7% decline in performance while β-alanine supplementation improved YoYo IR2 performance by ~34%, which compares favourably with the effects of pre-season training, and exceeds the expected CV of the test. Furthermore, all 8 of the players who improved with β-alanine did so above this expected CV, while the placebo group showed more variation with 3 players exceeding the CV (1 improved and 2 decreased their performance), which suggests that performance improvements in the β-alanine group can be attributed to the nutritional intervention employed in the current investigation. In addition, 4 of the players in the β-alanine group improved above the highest improvements shown following a 6–8 week training period (+45%; [12]). Since all players were involved in an identical training structure throughout the supplementation period, the further increases in these subjects could be attributed to an increased ability to train due to increased muscle buffering capacity [7], providing an additive effect over supplementation alone.

We chose to supplement amateur footballers during a competitive season as the YoYo IR2 has been shown to be sensitive to seasonal variation (CV: 14%; [13]) with scores, on average, lower during the season than at the start. Although mid-season scores were not different from the start of the season for First Division Scandinavian footballers, YoYo IR2 performance was decreased at the end of the season compared to the start of the season in another group of First and Second division players [13]. Furthermore, only 4 out of 15 players improved their YoYo IR2 performance during the season, while a further 9 showed a performance decrement [13]. In the present investigation, performance for players in the placebo group supplemented from early to mid-season followed a similar pattern to this, and all 3 supplemented from the middle until the end of the season showed a decline in performance. In contrast, all players supplemented with β-alanine from early- to mid-season improved their YoYo scores, while 2 of the 3 supplemented from mid-season until the end of the season showed a performance improvement, with the remaining player unchanged. These data provide evidence to suggest that β-alanine supplementation can not only halt the decline in fitness levels shown during a competitive season[13], but may even improve them above typical levels.

## Conclusions

The ingestion of 3.2 g·d^-1^ β-alanine over 12 weeks improved YoYo IR2 performance in amateur footballers during a competitive season. Improvements can be attributed to an increase in muscle buffering capacity due to increased muscle carnosine concentration, attenuating the decline in intramuscular pH during repeated high-intensity exercise bouts.

## Competing interests

We declare that we received β-alanine and maltodextrin supplies from Natural Alternatives International to undertake this study, though no additional funding was provided.

Roger Harris is an independent paid consultant of Natural Alternatives International, is named as an inventor on patents held by Natural Alternatives International, and is in receipt of other research grants awarded by Natural Alternatives International.

## Authors’ contributions

BS participated in the design of the study, carried out the data collection, performed the statistical analyses and drafted the manuscript. CS conceived of the study, participated in its design and helped draft the manuscript. RCH helped to draft the manuscript. CS conceived of the study, participated in its design and helped draft the manuscript. All authors read and approved the final manuscript.
